# Ten simple rules for organising an effective student-led writing retreat

**DOI:** 10.1371/journal.pcbi.1014147

**Published:** 2026-04-13

**Authors:** Nicholas W. Daudt, Claudia Hird, Eleanor R. M. Kelly, Elli E. Leinikki, Gretchen J. McCarthy, Ian S. Dixon-Anderson, Jackson E. Beagley, Jessica B. Moffitt, Joseph S. Curtis, Lindsay M. Wickman, Meghan L. Duffy, Preston L. Maluafiti, Saskia E. Foreman, William Carome, Leah M. Crowe

**Affiliations:** 1 Department of Marine Science, University of Otago, Dunedin, New Zealand; 2 Department of Mathematics and Statistics, University of Otago, Dunedin, New Zealand; 3 Department of Zoology, University of Otago, Dunedin, New Zealand; 4 Department of Geology, University of Otago, Dunedin, New Zealand; Carnegie Mellon University, UNITED STATES OF AMERICA

## Introduction

At every stage in a researcher’s career, scholarly output advances scientific knowledge and supports career development. Early career researchers, in particular, significantly boost their career prospects by increasing their scholarly outputs [1,2]. Writing serves as an integral skill for academic work [3,4], especially when competing for grants and jobs. Academics juggle administrative tasks alongside teaching, collection and analysis of data, and production of publications and presentations. Consequently, many report a lack of time to think critically as a major challenge in academia [5], which often leads researchers to deprioritise writing tasks [3,5], or, more recently, turn to generative artificial intelligence tools to tackle academic workloads [6]. Therefore, to fully engage in the act of writing, many need to fully disengage from other tasks by carving out dedicated focus time [7,8].

Writing retreats provide structured periods where researchers dedicate time to focused writing [7,9]. These retreats offer practical opportunities to disconnect from daily work routines [8,10], which help researchers gain writing momentum and increase scholarly output [9,11,12]. In addition, writing retreats foster a sense of community, promote well-being, and build self-confidence for academic writers [10–13]. Postgraduate students, in particular, highlight the value of these retreats in strengthening bonds among peers, obtaining and providing constructive feedback, and dedicating time and space to focus on writing [10,11,14,15].

A writing retreat was first proposed in 2023 by a PhD student in the Department of Marine Science at the University of Otago (Aotearoa/New Zealand) with the intention to not only facilitate community building within our PhD cohort [16,17], but also to structure a productive week as we worked toward our dissertation goals [9,11]. In New Zealand, PhDs follow research-only programs; as such, we do not participate in coursework that might promote group cohesion, as each student leads their own research. In addition, our diverse disciplines, field sites, and lab locations present challenges in maintaining social cohesion within our programme. As a cohort of PhD students, we organized a 5-day writing retreat in consultation with the Higher Education Development Centre at our university to be held at a remote field station. The retreat was a success both in terms of writing produced and connections built between peers sharing a similar journey. The retreat’s success motivated us to organise a second retreat the following year; the second retreat’s success inspired a third.

All retreat planning and implementation was conducted by PhD students. Planning, logistics, schedule creation, and end of retreat-reporting were led by one to three participating students each year, with input from all attendees. We present Ten Simple Rules for organising effective student-led writing retreats to share our experience in this process and to acknowledge the support of our academic department. Although the authors were all PhD students at the time they organised and participated in the retreats, these rules can be applied broadly to any research-oriented or academically-minded group. We outline steps to support the planning and execution of pre- (Rules 1–4), during (Rules 5–9), and post- (Rule 10) writing retreat actions, but we do not cover writing techniques per se. Many helpful resources on academic writing exist (e.g., [18–21]), including articles in this series [22–25].

## Rule 1: Find a venue

A change of scenery inspires productivity [8], whether you organise the writing retreat locally or as a multi-day trip [12]. When assessing suitability of the location, also consider internet access, heating/cooling, sleeping arrangements, needed accommodations for participants with particular needs, and workspace infrastructure (power points, seats, tables, etc.). For academics, university facilities could be leveraged. Taking advantage of institutional resources simplifies the planning process and enhances the success of your writing retreat. Local facilities, such as meeting rooms and other bookable spaces on campus, offer accessible options. For longer retreats, students—regardless of departmental affiliation—should inquire about their university’s field stations. For example, many biology and ecology programmes maintain field stations (for a global list, see [26]). By using university-owned facilities, students can likely keep retreat costs manageable.

Field stations often remain underutilized during certain periods, so retreats during these times can be used to promote year-round upkeep of facilities. In addition, field stations often sit in remote, natural settings with limited distractions, creating ideal environments for focus and uninterrupted work [8,10]. If your university lacks a suitable field station, other institutions may allow shared use depending on availability. We encourage students to contact station managers and build those connections.

For our writing retreat, we made use of our department’s marine field station in Oban, Rakiura/Stewart Island (a small island off the southern coast of New Zealand’s South Island). This location suited us well—everything we needed was within walking distance. Because this is a small community (around 400 year-round residents) abutting a national forest, we had limited distractions, straightforward options for restaurants and groceries, and ample access to walking tracks and beaches. For us, this choice of venue restricted how many people could attend as the field station is small (maximum capacity of 12 people). We have prioritised finding a venue in our list as it influenced all other details of the planning process.

## Rule 2: Propose a plan

Begin planning your retreat by preparing a proposal that outlines the intended structure and gauges interest from participants. Clearly define the capacity and target audience to plan the details of the retreat effectively. Your options for facilities (see Rule 1) may determine your maximum number of participants. In our case, we hosted all retreats with 10 participants; we found this number worked well for the size of our facility and the length of the retreat.

After outlining potential locations, dates, and the number of attendees, calculate the associated costs. These may include transportation, food, accommodation, and amenities such as internet.

Use the proposal to seek financial and in-kind support. Academic researchers may be able to access departmental or divisional resources (e.g., retreat space, vehicles for travel, and funding) to support opportunity for academic development. There may also be external funding available to provide supplemental support if internal money is not available to cover the entire costs of the retreat. Industry and community organisations often support academic pursuits, particularly when the benefits are felt local [27]. Another alternative is actively fundraising. A well-developed proposal lays a solid foundation for organising the retreat and securing the support needed to pull it off.

## Rule 3: Structure your retreat

Create a schedule for your writing retreat, as it provides the structure for focused writing and thinking time [7,10]. Use available resources to guide your planning (e.g., [10,28]), including this guide (see also Appendices). We collaborated with the Higher Education Development Centre at the University of Otago, which provided guidance on designing productive days and structuring the week for our first and second retreats.

The first and last day of the schedule were dedicated to travelling to and from the retreat location, allowing for the 5 days in between to remain fully structured for the retreat activities. We built in extra time on travel days to settle in, pack up, shop for groceries, and explore the area. Upon arrival, we held a welcome discussion facilitated by retreat leaders to establish ground rules to ensure everyone felt comfortable and had a shared understanding of expectations (see Rule 5). Each work day was themed to provide guidance and structure for participants (Table 1), while allowing each individual to pursue their own goals. We also incorporated optional social activities throughout the week (see Rule 9), such as a pub quiz, wildlife tours, game night, and local events.

**Table 1:** Examples of daily themes over a 5-day writing retreat. Note that days 1 and 7 were organised for travel.

Day	Theme	Description
Day 2	Getting started	Commit to the goal for the week (see Rule 4), organise a strategy, and establish accountability partners.
Day 3	Words on the page	Just start writing something! (Rule 6)
Day 4	Keep going!	Continue writing and refine. (Rule 6)
Day 5	Good enough	Exchange work within accountability partners to practice giving and receiving feedback. Use this opportunity to reflect on writing structure and style from a reader’s perspective. (Rule 8)
Day 6	Wrap it up	End the retreat by reflecting on what was accomplished this week (Rule 10) and develop a plan on how to proceed.

Our daily schedule typically ran from 9:00 AM to 6:00 PM and was shared in advance so participants knew what to expect during the week [10] (see also S1 Appendix). A different attendee volunteered to facilitate each day by leading discussions and managing writing periods (60–120 min) and breaks (30–60 min). Each morning began with a 15-min writing session, using themes that ranged from reflective writing to humorous research titles (see Rule 9). These sessions were used as a warm-up activity for the day and offered a fun, low-pressure opportunity to share writing. Each evening, the day was closed with a reflective discussion on progress made toward set goals with an ‘accountability partner’ (see Rule 5 in [24]), a shared dinner, and presentations (see Rule 7).

## Rule 4: Have a pre-retreat meeting

A pre-retreat meeting with all attendees gives participants a chance to meet, finalise logistics, set goals, and raise questions or concerns in advance. This time can be used to build or reinforce a respectful and accountable group dynamic (see Rules 5 and 10), and collaboratively fine-tune logistics and schedules. Most importantly, a pre-retreat meeting allows participants to identify or set specific writing goals while there is still time to prepare relevant resources (literature, data analysis, and input from academic supervisors). A pre-retreat meeting can help to stimulate participants to organise themselves before leaving so that nonwriting activities, like gathering references, do not become distractions during the initial days of the retreat.

We suggest that participants arrive at the pre-retreat meeting with a preliminary goal to share with the group. Goals may be quantitative (e.g., word count, page number) or qualitative (e.g., specific sections or revisions). For reference, during our 5-day retreats, participants averaged 1.2 pages or 550 words per day, working on everything from detailed revisions to drafting full manuscripts. By identifying a specific writing target ahead of time, friction related to making decisions is reduced during the retreat, with goals clear in mind. Sharing goals also helps participants learn about each other’s research and assess whether goals are realistic.

The pre-retreat meeting should be held within two weeks of departure to support timely preparation while preserving participant momentum. If possible, a casual, in-person meeting is preferred to provide a comfortable setting for participants to meet and connect. Nevertheless, if the majority of participants are away from campus or prefer an online meeting, this should be considered. Additional objectives may include selecting writing themes for the initial morning sessions, presentation topics, icebreakers, roles (e.g., meal coordination, daily facilitator), or ideas for social activities.

## Rule 5: Establish ground rules

Establish clear ground rules and expectations at the start of the retreat once you have arrived at the venue. These include shared expectations for the group, such as setting quiet hours, assigning household responsibilities (e.g., cooking and cleaning), and setting guidelines for breaks with consideration to the physical space. Ground rules should also set the tone of the retreat, creating a balanced foundation for respect, productivity, and enthusiasm.

We were encouraged to establish ground rules for our retreat by our university’s Higher Education Development Centre to have a shared agreement for behaviour to provide a safe and productive week. We prioritised respectful and nonjudgemental interactions between all participants. This attitude allowed flexibility in goal setting and accomplishments, depending on each person’s needs and stage of their PhD. Our baseline expectation of respect allowed us to set clear expectations for working versus quiet hours and to maintain a productive and peaceful environment. During our ground rule meeting, we also discussed the need for flexibility to ensure our retreat catered to the needs of all participants. For example, not all participants were native English speakers and full days of writing and discussion in English were more tiring. As a result, participants were encouraged to take the breaks they needed. Altogether, clear expectations, a set schedule, and an overall attitude of respect set the tone for a successful week of writing.

The document we used as the basis for our ground rules is in S2 Appendix.

## Rule 6: Write

Write. This is why you organise and participate in a writing retreat in the first place—to make progress on academic writing. The daily schedule (Rule 3) provides the structure to work toward your established writing goal (Rule 4), so it is time to execute. Knowing what you are working towards, and having a plan to get there will allow you to put words on the page and have productive writing periods.

However, writing is not easy, and writers face many hurdles, such as a lack of motivation, uncertainty about what to do next, writer’s block, imposter syndrome, and more [3,8,29,30]. These obstacles can easily disrupt even the best-laid plans and most robust retreat structures. Therefore, have a backup plan. If it becomes challenging to write, consider taking a break or switching tasks; it is proven that even short breaks increase productivity [20,31]. Everyone has different strategies that work for them. For example, during our retreats, some people preferred to cite as they wrote, while others wrote thoughts quickly and added citations later during editing. Some authors, such as Ernest Hemingway, have suggested to leave a sentence, paragraph, or idea unfinished at the end of a writing day so they have something easy to start with during the next writing period [32]. Assess what works best for your writing practice and reevaluate regularly to ensure that a strategy is still serving you [24,30].

## Rule 7: Lead an academic discussion

Schedule academic discussions around participants’ work in a structured manner. A writing retreat gathers your academic peers in a collaborative environment with minimal external distractions. It presents a great opportunity to garner advice and perspectives on written work (see also Rule 8) or any work-in-progress that participants may be developing. We suggest scheduling these discussions after writing periods are complete for the day and spreading them out over the retreat. Access to a projector and screen in our second and third retreats was beneficial. However, previously, we have simply shared from our laptop screens or consulted notes and found it equally engaging due to the small group attending the retreats. Discussions were informal, and we aimed not to exceed 20 min per session. It is important to emphasise the casualness of such an exercise—students should not anxiously prepare a conference-level presentation at the expense of their writing time. These are simply opportunities to share, learn, and exchange insights.

While we found academic discussions materialised naturally throughout the retreats, especially within groups of similarly focused researchers, we also allocated a small period of time each day (see Rule 3) specifically for students to share a talk or lead a discussion with the other attendees. The casual, nonjudgemental environment allowed presenting students a chance to practice a seminar talk, gain insight on methodologies, analyses and results, or simply share ‘tips and tools of the trade.’ Undertaking any large-writing project (e.g., a PhD thesis) is a challenging and variable experience. It is easy to become so engrossed in your work that you forget you are surrounded by people in a similar situation [29]. A writing retreat provides the opportunity to share your own research or discuss scientific/academic topics amongst your peers. Importantly, these sessions offer a chance to get to know one another and connect as fellow PhD students [16,17], aiming to combat a common feeling of loneliness during postgraduate research [33–35].

## Rule 8: Review and be reviewed

Schedule time to exchange written material and review the work of other participants. An essential aspect of developing your writing is critical review both in terms of giving and receiving feedback as well as through editing your own work [36,37]. Feedback from peers may offer valuable insights on gaps in clarity, logic, and structure. Even if you do not understand the scientific content of the work, focusing on structure, prose, and writing flow can be extremely helpful. This type of review can unblock a colleague who feels stuck. Likewise, feedback from a peer outside one’s field may offer perspectives the author had not previously considered.

Providing feedback is just as valuable as receiving it. This practice encourages a collaborative approach to writing that builds confidence and resilience in both the reviewer and the reviewed. We were provided with guidance for offering constructive feedback, focusing on writing components such as structure, clarity, and flow. Neutral language allows a reviewer to remain objective by avoiding using words that express feelings about the content (i.e., avoid using words like ‘unfortunately’, ‘surprisingly’, ‘failed’, ‘forgot’, etc.) [38]. If you are an expert in the subject matter, detailed feedback on the content can also be extremely valuable.

Peer review is an integral part of academic life [39], and informal review among students provides an opportunity to practice giving constructive feedback. Furthermore, researchers should be mindful of the historical barriers peer review imposes on minority groups and non-English speakers [40,41], and work towards breaking this pattern. A writing retreat is a friendly, safe space to talk about these barriers (Rule 7) and become more comfortable with the review process in general while practising writing critical, neutral, and encouraging feedback. Our retreat schedule incorporated periods to exchange and review writing toward the end of the week (Table 1) to give participants time to make progress on writing, but also time to incorporate feedback before the retreat ended.

## Rule 9: Have fun

Have fun and build connections with your peers. While on a writing retreat, recreational activities can enhance the overall experience. Furthermore, balancing downtime activities with writing periods can lead to optimal productivity [13,31], improving the writing experience. A writing retreat allows you to disconnect from regular routines and responsibilities [8], providing a unique opportunity to bond with your peers. Rural places often have scenic landscapes, hospitable communities, and limited distractions. While the choices for activities may be limited, a rural setting can offer a slower pace and can lead to high-quality experiences getting to know local businesses and other retreat attendees on a more personal level. Before embarking on a writing retreat, research what the location has to offer and take advantage of the opportunities available.

We used our university’s field station based on Rakiura/Stewart Island for our retreat, an island known for its small town, National Park, dark sky sanctuary, and unique New Zealand bird life. In the evenings, we offered optional opportunities as part of our writing retreat schedule to explore the area as a group, including a trip to the nearby Ulva Island (a protected area), participation in the locally famous pub quiz, and looking for the aurora and kiwi birds at night. In addition, we organised creative writing themes each morning (see Rule 3; some were hosted at the local cafe), which created fun moments to start the day. The casual presentations (Rule 7) about various topics were fun and engaging bonding activities, allowing us to connect deeper with each other through our work.

## Rule 10: Reflect, document, and acknowledge

After the retreat, take time to reflect and gather feedback from participants. A group discussion about the retreat’s highlights and challenges may help clarify thoughts before participants are asked to complete an anonymous survey. Shortly after returning, summarise the feedback into a concise report while the details are still fresh. Be sure to include feedback on the schedule and notes on aspects that went well, including benefits of the retreat, or poorly to address in future retreat planning. This is valuable for cementing ideas and details that would otherwise fade before another retreat can be planned. These insights will shape future retreats and ensure important lessons are not lost over time.

A report also creates accountability and continuity. The ritual of compiling information about the retreat not only provides space to record a list of participants and their achievements, but also to responsibly give an account to those who supported the initiative. A summary report from each retreat was presented to staff in our Department, who provided financial and logistical support. The report offered an opportunity to share details of our retreat planning and execution, writing accomplishments, challenges, recommendations for future retreats, and pictures of the participating group. Furthermore, student organisers will inevitably change over time, but a shared archive of resources—such as schedules, ground rules, and facilitation tips—makes it easier to plan future, effective events. Creating an institutional repository for retreat materials provides blueprints and templates to future organisers, setting them up for success.

Finally, publicly acknowledging the retreat in your writing and presentations demonstrates its academic value. Participants are encouraged to include the retreat in the acknowledgements of manuscripts and theses worked on during the retreat. Sharing this impact helps advocate for continued support, especially in institutions where publication output informs resource allocation. Maintaining a list of retreat-supported outputs can strengthen future proposals (see Rule 2) and make a compelling case for funding. Indeed, demonstrating the immense value of this experience may spur future support from leadership to fund retreats for students in other departments or institutions.

## Conclusion

Collectively, we experienced many of the benefits of writing retreats reported in the literature. These included feeling empowered as writers through supporting each other [14], increasing our sense of belonging as PhD students [16], and decreasing our sense of isolation [13]. Recent literature has reported that many of these same challenges may be exacerbated in postgraduate programs as reliance on artificial intelligence tools becomes more common than leaning on peers [42]. Here, we add to the literature another example of how convening with peers to share in writing challenges yields net benefits for academic communities. Participants came away from each retreat as better friends and colleagues, which fostered a culture of belonging within the Department, and led to collaborative organisation of other events (e.g., a postgraduate symposium). Most importantly, we achieved writing success during these retreats as participants returned to campus with new pages in hand. Some of the references herein can help with the theoretical side of writing, but writing is critical thinking [43,44], and getting better at it ultimately requires practice and dedicated time [24,30]. By facilitating the planning and execution of writing retreats through these Ten Simple Rules, we hope to foster scholarly productivity and well-being in student and early career cohorts. Happy writing!

## Supporting information

S1 Appendix Schedule Examples. Daily schedules used during our writing retreats in 2023, 2024, and 2025.
(PDF)

S2 Appendix Ground Rules. The base document for the Ground Rules was discussed at the start of the retreats. Any additional points can be added to tailor the specifics of each group/location.
(PDF)
