# Supplementary material for: Ten simple rules for organising an effective student-led writing retreat
Source: PLoS Comput Biol. 2026 Apr 13;22(4):e1014147. doi: 10.1371/journal.pcbi.1014147 (PMC13075675; doi:10.1371/journal.pcbi.1014147)
Supplement: S1 Appendix — Daily schedules used during our writing retreats in 2023, 2024, and 2025. (PDF) [file pcbi.1014147.s001.pdf]

PLoS Computational Biology

Ten simple rules for organising an effective student-led writing retreat

Nicholas W. Daudt*, Claudia Hird, Eleanor R. M. Kelly, Elli E. Leinikki, Gretchen J. McCarthy, Ian S. Dixon-Anderson, Jackson E. Beagley, Jessica B. Moffitt, Joseph S. Curtis, Lindsay M. Wickman, Meghan L. Duffy, Preston L. Maluafiti, Saskia E. Foreman, William Carome, Leah M. Crowe

* nicholaswdaudt@gmail.com

==

Schedule Examples

2023

	Day 1	Day 2	Day 3	Day 4	Day 5	Day 6	Day 7
**08:00**	Travel	Breakfast	Breakfast	Breakfast	Breakfast	Breakfast	Breakfast/pack up
**09:00**	Travel	Writing exercise	Writing exercise	Writing exercise	Writing exercise	Writing exercise	Breakfast/pack up
**09:15**	Travel	Morning activity	Discussion	Discussion	Discussion	Discussion	Breakfast/pack up
**09:30**	Travel	Write	Write	Write	Write	Write	Breakfast/pack up
**10:00**	Travel	Break	Break	Break	Break	Break	Group activity
**11:00**	Settle in/ Ground rules	Write	Write	Write	Write	Write	Group activity
**12:00**	Lunch	Lunch	Lunch	Lunch	Lunch	Lunch	Group activity
**13:00**	Free time	Write	Write	Write	Review/editing	Write	Group activity
**13:30**	Free time	Write	Write	Write	Review/editing	Write	Group activity
**14:00**	Free time	Write	Write	Write	Review/editing	Write	Group activity
**15:00**	Free time	Break	Break	Break	Break	Break	Travel
**16:00**	Free time	Write	Write	Write	Write	Write	Travel
**17:00**	Dinner	Acountability check	Acountability check	Acountability check	Acountability check	Acountability check	Travel
**18:00**	Group activity	Stop	Stop	Stop	Stop	Stop	Travel
**18:30**	Group activity	Dinner	Dinner	Dinner	Dinner	Dinner (out)	Travel
**19:30**	Group activity	Dinner	Dinner	Dinner	Dinner	Dinner (out)	Travel
**20:00**	Group activity	Presentation	Presentation	Free time	Presentation	Presentation	Travel
	Free time	Free time	Free time	Free time	Free time	Free time	-

2024

	Day 1	Day 2	Day 3	Day 4	Day 5	Day 6	Day 7
**08:00**	Travel	Breakfast	Breakfast	Breakfast	Breakfast	Breakfast	Breakfast/pack up
**08:45**	Travel	Writing exercise	Writing exercise	Writing exercise	Writing exercise	Writing exercise	Breakfast/pack up
**09:00**	Travel	Write	Write	Write	Write	Write	Breakfast/pack up
**10:30**	Travel	Break	Break	Break	Break	Break	Group activity
**11:00**	Settle in/ Ground rules	Write	Write	Write	Write	Write	Group activity
**12:30**	Lunch	Lunch	Lunch	Lunch	Lunch	Lunch	Group activity
**13:30**	Free time	Write	Write	Write	Write	Write	Group activity
**15:00**	Free time	Break	Break	Break	Break	Break	Travel
**16:00**	Free time	Write	Write	Write	Review/editing	Review/editing	Travel
**17:00**	Dinner	Acountability check	Accountability check	Accountability check	Accountability check	Accountability check	Travel
**17:15**	Dinner	Presentations	Presentations	Presentations	Presentations	Presentations	Travel
**18:00**	Group activity	Stop	Stop	Stop	Stop	Stop	Travel
**18:30**	Group activity	Dinner	Dinner	Dinner	Dinner	Dinner (out)	Travel
**19:00**	Group activity	Dinner	Dinner	Dinner	Dinner	Dinner (out)	Travel
**19:30**	Group activity	Dinner	Group activity	Dinner	Dinner	Dinner (out)	Travel
	Free time	Free time	Free time	Free time	Free time	Free time	-

2025

	Day 1	Day 2	Day 3	Day 4	Day 5	Day 6	Day 7
**08:00**	Travel	Breakfast	Breakfast	Breakfast	Breakfast	Breakfast	Breakfast/pack up
**09:00**	Travel	Writing exercise	Writing exercise	Writing exercise	Writing exercise	Writing exercise	Breakfast/pack up
**09:30**	Travel	Write	Write	Write	Write	Write	Group activity
**10:30**	Travel	Break	Break	Break	Break	Break	Group activity
**11:00**	Settle in	Write	Write	Write	Write	Write	Group activity
**12:00**	Lunch	Lunch	Lunch	Lunch	Lunch	Lunch	Group activity
**13:00**	Lunch	Write	Write	Write	Review/editing	Write	Group activity
**13:30**	Lunch	Write	Write	Write	Review/editing	Write	Group activity
**15:00**	Free time	Break	Break	Break	Break	Break	Travel
**15:30**	Free time	Write	Write	Write	Write	Accountability check	Travel
**16:00**	Free time	Write	Write	Write	Write	Presentations*	Travel
**17:30**	Ground rules	Accountability check	Accountability check	Accountability check	Accountability check	Presentations*	Travel
**18:30**	Dinner	Dinner	Dinner	Dinner	Dinner	Dinner (out)	Travel
**19:30**	Dinner	Dinner	Dinner	Dinner	Dinner	Dinner (out)	Travel
	Free time	Group activity	Free time	Free time	Free time	Group activity	-

* We concentrated the presentations at the end of the last day of the retreat this year. However, it was not the most effective strategy, according to feedback from participants. Therefore, as stated in the main text, we recommend spreading them out across the writing retreat.
