# Supplementary material for: Ten simple rules for organising an effective student-led writing retreat
Source: PLoS Comput Biol. 2026 Apr 13;22(4):e1014147. doi: 10.1371/journal.pcbi.1014147 (PMC13075675; doi:10.1371/journal.pcbi.1014147)
Supplement: S2 Appendix — The base document for the Ground Rules was discussed at the start of the retreats. Any additional points can be added to tailor the specifics of each group/location. (PDF) [file pcbi.1014147.s002.pdf]

PLoS Computational Biology

Ten simple rules for organising an effective student-led writing retreat

Nicholas W. Daudt*, Claudia Hird, Eleanor R. M. Kelly, Elli E. Leinikki, Gretchen J. McCarthy, Ian S. Dixon-Anderson, Jackson E. Beagley, Jessica B. Moffitt, Joseph S. Curtis, Lindsay M. Wickman, Meghan L. Duffy, Preston L. Maluafiti, Saskia E. Foreman, William Carome, Leah M. Crowe

* nicholaswdaudt@gmail.com

==

Ground Rules

- Lab space is writing space.
- Sleeping space is sleeping space.
- No partying in the field station, quiet after 10:00 PM, unless everyone is still up.
- Put down words. This is a writing retreat, so write.
- Respect that English isn't everyone's first language, but that doesn't matter: Just write/Ecrivez/Apenas escreva/Kirjoita/Solo escribe/נאָר שרײַבן/Tuhituhi noa!
- Respect the clock. If you can't get in the right head space, take a walk.
- Respect each other. Kind, constructive criticism only!
- Be punctual for group discussions and meals.
